# Altered gene expression in asymptomatic SHIV-infected rhesus macaques (*Macacca mulatta*)

**DOI:** 10.1186/1743-422X-3-74

**Published:** 2006-09-06

**Authors:** Erica E Carroll, Rasha Hammamieh, Nabarun Chakraborty, Aaron T Phillips, Stacy-Ann M Miller, Marti Jett

**Affiliations:** 1 Division of Pathology, Walter Reed Army Institute of Research, Silver Spring, Maryland, USA

## Abstract

Simian-Human immunodeficiency virus is a chimeric virus which, in rhesus macaques (*Macacca mulatta*) closely imitates immunodeficiency virus infection in human (HIV). A relatively new way to study pathogenesis of viral infection is to study alterations in host gene expression induced by the virus. SHIV infection with certain strains does not result in clinical signs. We hypothesized that alterations in gene expression relating to the immune system would be present in SHIV-infected animals despite the lack of clinical signs. Splenic tissue from four adult male Indian-origin Rhesus monkeys serologically positive for non-pathogenic SHIV 89.6 was processed by cDNA microarray analysis. Results were compared with the corresponding outcome using splenic tissues from four unexposed adult male Rhesus monkeys. Subsequent gene analysis confirmed statistically significant variations between control and infected samples. Interestingly, SHIV-infected monkeys exhibited altered expression in genes related to apoptosis, signal transduction, T and B lymphocyte activation and importantly, to immune regulation. Although infected animals appeared asymptomatic, our study demonstrated that SHIV-infected monkeys cannot reliably be used in studies of other infectious agents as their baseline gene expression differs from that of normal Rhesus monkeys. The gene expression differences in SHIV-infected animals relative to uninfected animals offer additional clues to the pathogenesis of altered immune function in response to secondary infection.

## Background

Simian immunodeficiency virus (SIV) infection of rhesus macaques exhibits many similarities to human immunodeficiency viral (HIV) infection of humans. Most pathogenesis and vaccine studies for HIV-1 have been undertaken in either SIV-macaque or a chimeric simian-human immunodeficiency (SHIV)-macaque model [1]. SHIV strains have the viral envelope of HIV but the gag/pol genes of SIV. Pathogenesis is similar with respect to macrophage and T lymphocyte cell tropism, histopathologic changes, CD4-cell depletion and clinical signs of auto-immune deficiency syndrome (AIDS) in virulent strains. HIV and SIV additionally cause cognitive and motor impairments in infected patients and monkeys, respectively [2]. Host factors may play a role in degree of pathogenesis between varying SHIV constructs, as one study reported observing similar viral loads in rhesus monkeys infected with pathogenic and non-pathogenic SHIV constructs [1].

Gene expression studies have grown increasingly popular as a tool to mine large amounts of data from treated and control populations. Such data can be used to examine host factors involved in SHIV, and thereby HIV, pathogenesis. To our knowledge, microarray data from SHIV-infected Rhesus macaques have not yet been examined for genes affecting immune response and inflammation. Gene expression data have the potential to greatly expand the understanding of SHIV-host interaction beyond the limited number of cell types or cytokines generally examined.

In animals free of clinical signs of SHIV, altered baseline gene expression data may give clues to the pathogenesis of altered immune response to secondary infections. Studies involving HIV-infected humans demonstrated suppression of IL-2 in response to select antigens and increase in TNF-α even prior to the onset of CD4+ T-cell depletion [3,4]. Gene expression data collected in this study from SHIV 89.6-infected monkeys demonstrate that these animals are not genetically 'normal' and cannot ethically be used for studies involving other infectious agents, if at all, without an explicit caveat listing their SHIV status. Comparison of gene expression patterns collected from SHIV-infected and uninfected animals to that of the matched animals exposed to select bacterial and viral agents would provide a more complete understanding of SHIV effect on immune response to particular infectious agents. Extrapolation to the HIV-patient response to secondary agents may then be attempted. Gene expression data may also provide clues to pathogenesis of cognitive and related ailments arising with HIV infection.

## Results

### Clinical history

A brief description of treated and control animals is given in Table 1. All monkeys were male; while two of them (one SHIV-positive, one SHIV-negative) were Herpes B-positive.

**Table 1:** An overview of the Rhesus macaques used in SHIV gene expression study

**Animal ID**	**Gender**	**Age (yrs)**	**Geographic origin**	**Herpes B Status**	**SHIV 89.6 status**
JGH	Male	7	Indian	positive	positive
PHB	Male	7	Indian	negative	positive
TTH	Male	7	Indian	negative	positive
FFG	Male	9	Indian	negative	positive
331	Male	adult	Indian	negative	negative
332	Male	adult	Indian	negative	negative
CJ2T	Male	4	Indian	negative	negative
EC49	Male	adult	Indian	negative	negative
DB87	Male	12.2	Indian	positive	negative

Table 2 summarizes abnormalities in clinical chemistries including complete blood counts of the SHIV-infected animals. Abnormalities were minimal. Attending veterinary clinicians considered these animals asymptomatic with respect to SHIV infection.

**Table 2:** Clinical pathology of SHIV-positive rhesus macaques

**Animal ID**	**Abnormal findings in complete blood count and serum chemistry analysis.**
**FFG**	Sodium 144 mg/dl (reference range 147–158) Chloride 108 mg/dl (range 110–120) Lymphocytes 65.4% (reference range 14–64%)
**PHB**	Sodium 146 mg/dl (range 147–158) Carbon dioxide 29 mmol/L (range 19–29) Total protein 6.4 g/dl (range 6.7–8.0) ALT 113 U/L (range 20–91) LDH 538 U/L (range 638–3012)
**TTH**	Sodium 145 mg/dl (range 147–158) Chloride 110 mg/dl (range 110–120) AST 29 U/L (range 29–64)
**JGH (Herpes B+)**	Sodium 147 mg/dl (range 147–158) Chloride 109 mg/dl (range 110–120) Carbon dioxide 30 mmol/L (range 19–29) Triglycerides 18 mg/dl (range 35–137) Total protein 6.6 g/dl (range 6.7–8.0) AST 26 U/L (range 29–64)

**Table 3:** The sequences of the primers used in the present project

**Name**	**Gene Bank ID**	**Description**	**Sequence**	**Product Size**
ANLN	R16712	Anilin	5'-TCC AAG TCC TGT GTC TCC TC-3'	
			5'-TCT TGA GTT CAG CCC TCT CC-3'	109 bp
Bit1	AI339248	CGI-147 protein	5'-TGG CTG TTG GAG TTG CTT G-3'	
			5'-TGT GTG TCT TGC TCG TCT TG-3'	93 bp
CLCA2	AI675394	chloride channel. calcium activated, fam	5'-CAA CCA AGA AGC ACC AA CC-3'	
			5'-CAT CCA GCA CTA AAC AGA CCA C-3'	179 bp
	AA922998	postmeiotic segregation increased 2-like	5'-GTT TCA GGC AAT GGA TGT GG-3'	
			5'-CAT GGC AGG TAG AAA TGG TG-3'	178 bp
COL15A	AA455157	collagen, type XV, alpha 1	5'-CCA CCT ACC GAG CAT TCT TAT C-3'	
			5'-CAA TAC GTC TCG ACC ATC AAA G-3'	197 bp
IL2RA	AA903183	interleukin 2 receptor, alpha	5'-CTG AGA GCA TCT GCA AAA TGA C-3'	
			5'-GGC CAC TGC TAC TTG GTA CTC T-3'	242 bp
PDCD4	N71003	programmed cell death 4	5'-CCG GTG ATG AAG AAA ATG CT-3'	
			5'-TGG TTG GCA CAG TTA ATC CA-3'	207 bp
ADORA2	N57553	adenosine A2a receptor	5'-TCA ACA GCA ACC TGC AGA AC-3'	
			5'-ATG GCA ATG TAG CGG TCA AT-3'	220 bp
RBM9	AA451903	RNA binding motif protein 9	5'-AAC TCC TGA CTC AAT GGT TC-3'	
			5'-CAT TTT GTG TGC TGG GTG AG-3'	194 bp
MAP2K7	H85962	mitogen-activated protein kinase kinase	5'-ACC AGG CAG AAA TCA ACG AC-3'	
			5'-GAT GAA CGT CCC AAA GCA CT-3'	224 bp
COL7A1	AA598507	collagen, tykpe VII, alpha 1 (epidermolysin)	5'-AGC CCA GAT GTT TCC ACT CA-3'	
			5'-ACA AGA GGC AAT CCT TGG AGA-3'	239 bp

### Micro-array analysis of SHIV-infected versus uninfected

Using the 38 most varying genes between SHIV-infected and SHIV-uninfected animals, we performed Principle Component Analysis, a non-hierarchal clustering tool, to revalidate the *t*-test result. Figure 2 demonstrates that the SHIV positive and negative groups were clustered together, keeping a significant distance between them along the first principal component (X-axis), which shared the highest fraction of group variation. The pattern of clustering also suggested that the gene expression variability was independent of the animals' Herpes B status.

**Figure 1:**
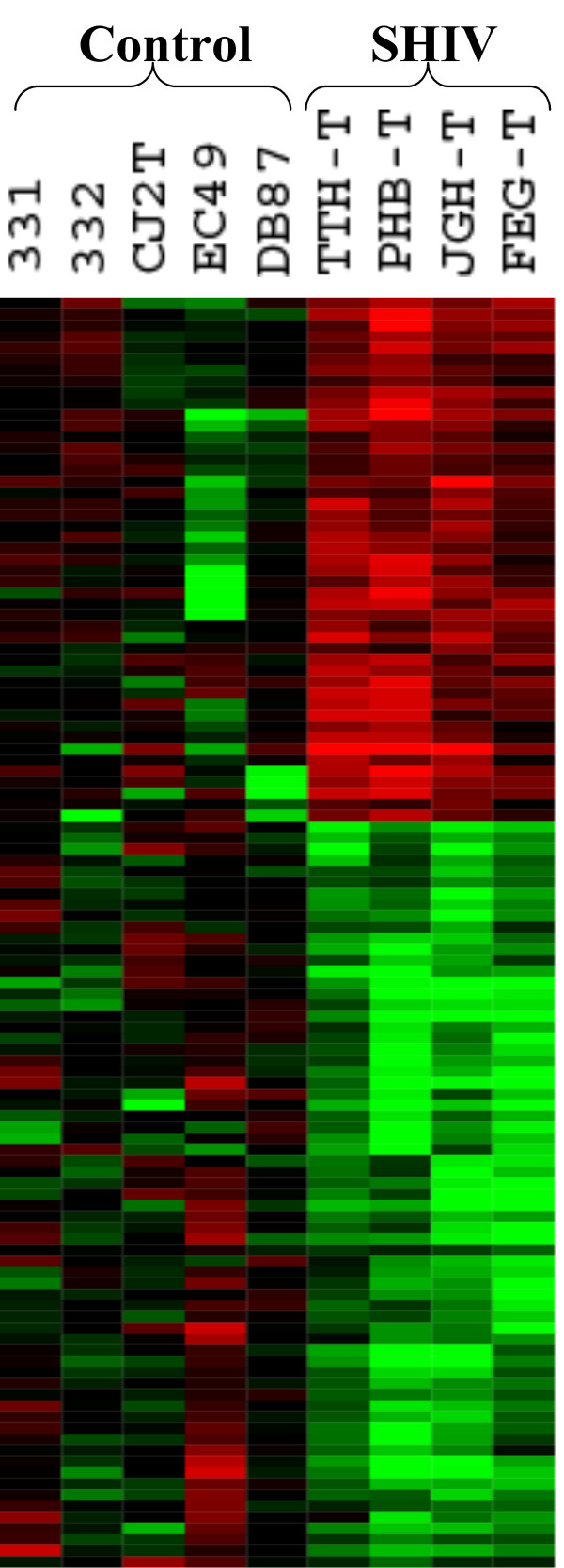
Hieratically clustered Tree-view of genes differentially expressed between the SHIV positive and negative animals.

**Figure 2:**
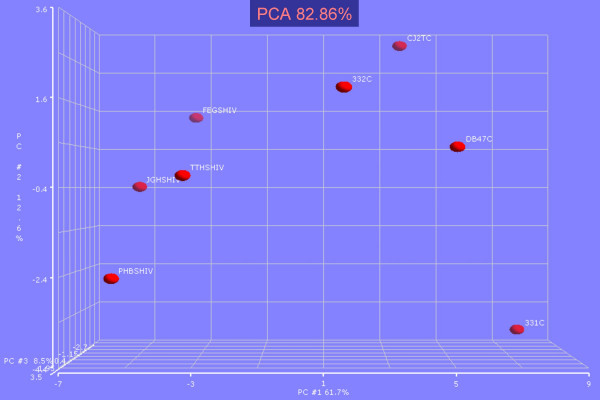
Principal component analysis was performed over the SHIV infected and non-infected population. Though the animals were clinically reported asymptomatic, the SHIV treated and control samples cluster far from each other along PCA1 axis. The result also suggests that the Herpes B status does not affect the outcome. Here PCA1 has 61.7% population, while PCA2 and PCA3 shares 12.6% and 8.56% of the population respectively.

Gene ontology study, using GeneCite [6], associated the members of the differentially expressed genes to a range of important biological and pathological functions including immune defense, cell death or apoptosis, cell growth, signal transduction and others. Table 4 represents the functional classification of some of the genes of interest.

**Table 4:** The list of some of the genes of interest.

**Gene ID**	**Symbol**	**Gene Name**	**Fold Change**
*Cellular defense immunity:*			
AA424786	GOLGA2	golgi autoantigen, subfamily a2	2.802126
AA664195	HLA-DRB3 (HLA-DRB1)	major histocompatibility complex, class II, DR beta 1	0.202677
AI815229	LILRB3	leukocyte immunoglobulin-like receptor, subfamily B, member 3	0.074432
H96643	FOSL1	FOS-like antigen-1	0.284931
			
*Cell growth/proliferation:*			
AA035384	SDHD	succinate dehydrogenase complex	0.287502
AA521228	HIBCH	3-hydroxyisobutyryl-Coenzyme A hydrolase	4.260302
AA699573	TCF2	hepatic transcription factor 2	4.223543
AI220577	TNP2	transition protein 2	0.262051
H06676	ALDH5A1	aldehyde dehydrogenase 5 family	2.381241
AI798238	P2RY11	peter pan homolog	0.174406
			
*Cell death/Apoptosis:*			
AA458838	NOXA	phorbol-12-myristate-13-acetate-induced protein 1	3.872641
AI339248	Bit1	CGI-147 protein	0.337378
AI972925	API5	apoptosis inhibitor 5	0.17877
			
*Molecular binding/Adhesion:*			
AA167269	NAP1L1	nucleosome assembly protein 1-like 1	0.272199
AA424824	DSTN	destrin	2.876146
AA669637	PNRC1	proline rich 2	0.142976
AA676840	UTRN	utrophin	2.340295
AI769340	HRC	histidine-rich calcium-binding protein	0.220777
R16712	ANLN	anillin	0.280955
T60070	RAB40B	GTP-binding protein, member RAS oncogene family	2.649082
AA426374	TUBA2	alpha tubulin 2	0.112352
AA055163	CASQ2	calsequestrin 2	0.531223
AA521350	Sep15	15 kDa selenoprotein	0.33198
AA633747	COL6A2	collagen, type VI, alpha 2	2.061697
AA634218	PRAF2	JM4 protein	0.35942
AA922998	PMS2L5	postmeiotic segregation increased 2-like 5	0.289763
AI364103	CINP	cyclin-dependent kinase 2-interacting protein	3.399017
AI653424	NUFIP1	nuclear fragile X mental retardation protein interacting protein 1	0.15423
W32272	IQGAP2	IQ motif containing GTPase activating protein	3.137166
			
*Signal Transduction:*			
AA427491	TRAC	T-cell receptor active alpha-chain	0.145492
AI401275	CALCR	calcitonin receptor	0.329203
AA421819	CDH6	K-cadherin	0.241252
			
*Transport:*			
AI675394	CLCA2	calcium activated chloride channel	3.802165
W94331	CTNS	nephropathic cystinosis	0.212335
N46828	ITPKC	inositol 1,4,5-trisphosphate 3-kinase C	5.969257
			
*Biogenesis:*			
AA056013	MAGP2	Microfibril-associated glycoprotein-2	2.312604
AA629189	KRT4	keratin 4	0.227523
H27864		secretogranin II	0.089345

The first, second and third columns list the GeneBank ID, Symbol and Gene Name respectively. The Fourth column stands for the corresponding fold change of SHIV positive animal with respect to that of the control animal, averaged over the entire population, i.e. (Average fold change for all SHIV positive animals)/(Avg FC for all control animals)

### Confirmation of gene expression changes by Real-Time PCR analysis

**Figure 3:**
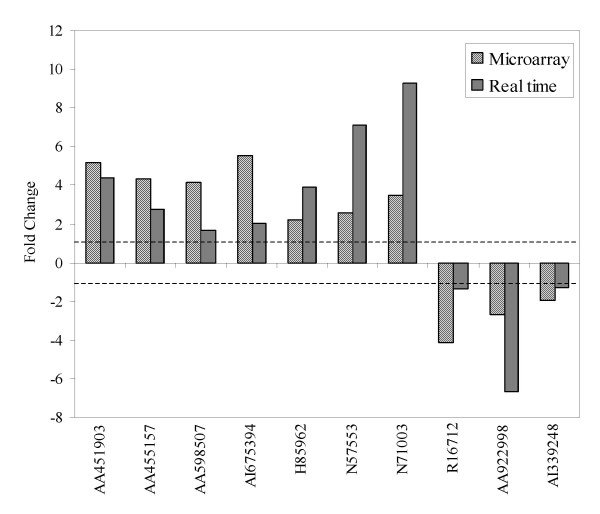
A comparative analysis of four selected genes using array analysis and Real-time PCR. RNA binding motif protein 9 (AA451903), collagen, type XV, alpha 1 (AA455157), collagen, type VII, alpha 1(AA598507), interleukin 2 receptor, alpha (AA903183), Chloride channel, calcium activated, family member 2 (AI675394), mitogen-activated protein kinase kinase (H85962), adenosine A2a receptor (N57553) and programmed cell death 4 (N71003) were up regulated in SHIV infected animals while postmeiotic segregation increased 2-like (AA922998), Bcl-2 inhibitor of transcription (AI339248) and Anillin (R16712) were down regulated.

Ten genes were selected for real-time polymerase chain reaction (PCR). They are RNA binding motif protein 9 (AA451903), collagen, type XV, alpha 1 (AA455157), collagen, type VII, alpha 1(AA598507), interleukin 2 receptor, alpha (AA903183), Chloride channel, calcium activated, family member 2 (AI675394), mitogen-activated protein kinase kinase (H85962), adenosine A2a receptor (N57553), programmed cell death 4 (N71003), postmeiotic segregation increased 2-like (AA922998), Bcl-2 inhibitor of transcription (AI339248) and Anillin (R16712). Figure 3 illustrates that the real-time PCR expression profiles for the selected genes are well correlated with the corresponding microarray results.

## Discussion

Simian immunodeficiency virus (SIV), previously referred to as simian T-cell lymphotropic virus type III (STLV-III), induces an AIDS-like disease in its natural host, rhesus macaques. HIV and SIV, members of the lentivirus subfamily of retroviruses, not only resemble each other by their antigenicity, but also bear remarkable similarity in their biological properties, such as cytopathic effect and tropism for CD4-bearing cells. These criteria render the chimeric SHIV the best animal model currently available for HIV study.

In this study, we examined gene expression in SHIV- infected male rhesus macaques of Indian origin using a genomic perspective and compared the results to uninfected age, gender and Herpes B-status-matched controls. Although infected animals were without clinical signs related to SHIV infection, a significant number of genes exhibited significantly altered expression concurrent with SIV infection.

Ontological research revealed that several genes, namely FOS-like antigen 1 (FOSL1, ID: H96643), golgi autoantigen (GOLGA2, ID: AA424786), major histocompatibility complex (MHC), class II, DR beta 1 (HLA-DRB1, ID: AA664195) and leukocyte immunoglobulin-like receptor (LILRB3, ID: AI815229) are associated with human immune defense. LILRB3 is a leukocyte inhibitory receptor which, upon binding to MHC Class I molecules, transmits inhibitory signals to the nucleus. HLA-DRB1, down regulated by SIV infection, is a cell-surface-associated immunoregulatory protein. Interestingly, this human leukocyte antigen (HLA)-associated gene has been correlated with non-responsiveness to recombinant hepatitis B virus (HBV) vaccine but does not alter susceptibility to viral persistence [6]. Another MHC protein binding unit, T cell receptor alpha locus (TRAC, ID: AA427491) is ontologically related to signal transduction.

Gene ontology investigation classified a significant subset of the genome of interest as a regulator of cell growth and apoptosis. SIV infection results in down-regulation of apoptosis inhibitor 5 (API5, ID: AI972925) and up-regulation of pro-apoptotic protein phorbol-12-myristate-13-acetate-induced protein 1 (NOXA, ID: AA458838) [8]. These alterations in gene expression might instigate opportunistic infections by inducing apoptosis among T-helper lymphocytes. Likewise, SIV infection alters several metabolism and cell growth regulating factors. For example, SIV-infected genome contains upregulated aldehyde dehydrogenase 5 family member A1 (ALDH5A1, ID: H06676); and concurrent down regulated succinate dehydrogenase complex, subunit D (SDHD, ID: AA035384) and nephropathic cystinosis (CTNS, ID: W94331). Reports suggest that overexpressed ALDH5A1 changes the concentration of gamma-aminobutyric acid (GABA) and glutamate, commencing henceforth excitotoxic damage, a well-established clinical marker of HIV activity [9]. Underexpressed SDHD and CTNS are associated with immunodeficiency through curbed monocyte and CD4+ T cell -induced immunoregulation [10], respectively.

Several entries of the present genome are functionally related to cellular and molecular transportation and binding. Interestingly, five actin-binding genes appeared in the list; namely: anillin (ANLN, ID: R16712), destrin (DSTN, ID: AA424824), utrophin (UTRN, ID: AA676840), cyclin-dependent kinase 2-interacting protein (CINP, ID: AI364103) and IQ motif containing GTPase activating protein 2 (IQGAP2, ID: W32272). Actin, the ubiquitously present cellular protein, has been reported to guide the direct cell-to-cell HIV-1 propagation by making of a stable adhesive junction at the target-effector cell interface [11]. Table 4 displays the down regulation of another molecular binding protein, 15 kDa selenoprotein (SEP15, ID: AA521350). Reduced level of selenoprotein in cells is a known marker of in vitro infection of SHIV [12]. Our data also supports the fact that immunodeficiency is correlated with altered calcium ion binding (UTRN, ID: AA676840; CDH6, ID: AA421819, CASQ2, ID: AA055163) and also is influenced by calcium- activated chloride channels (CLCA2, ID: AI675394) of host cells. Those are well established pathoregulating markers of activ HIV-1 negative factor (Nef) [13-15].

In summary, in this small sample of SHIV-infected Rhesus macaques, expression was consistently altered in specific groups of genes which regulate a broad range of biochemical functions. A few important members of the genome of interest are discussed here. The present study, along with correlating some genes with SHIV and HIV model, identifies several novel genes as potential therapeutic markers for immune deficiency studies. Furthermore, results of this study suggest that SHIV infection of rhesus macaques may influence immune response to a second agent, even if baseline levels of clinical measurements appear normal. This study substantiates and validates the concern that an infected (i.e., antibody-producing) but asymptomatic animal should not be used in any other study involving infectious agents unless the pattern of gene expression to that agent is compared to normal animals' pattern, one agent at a time.

Note: microarray data have been submitted to the Gene Expression Omnibus (GEO) and can be searched using the Platform ID: GPL3395.

## Materials and methods

### Animals and virus

Four adult (7–8 years old) male Rhesus macaques (one Herpes B-positive and three Herpes B-negative) that were previously exposed to SHIV 89.6 strain (Animal identifications: FFG, JGH, PHB and TTH) were euthanized due to being declared 'excess' and no longer usable due to their serologically positive SHIV status. Splenic tissue was collected from each animal upon euthanasia and immersed in RNA Later^® ^for 30–60 minutes before freezing at -80C.

SHIV 89.6, like all SHIV strains, has the *env *gene from the HIV-1 strain. All four animals had been challenged with 1.0 ml intravenous SHIV 89.6, a non-pathogenic strain, and became seropositive. Previous studies by the same researchers showed that seropositive animals were PCR positive as well (WRAIR Protocol TO03-98). All animals remained free of clinical signs. Complete blood counts and serum chemistry profiles were performed on the SHIV-positive animals and were within or very close to normal limits. The negative control animals were Indian-origin adult male Herpes B-negative Rhesus macaques. Splenic tissues were kindly provided by Scripps Institute, the National Institute of Health, and the Oregon National Primate Research Center. Tissue from a SHIV-negative animal (DB-87, provided by the Tulane National Regional Primate Research Center) was Herpes B-positive to control for the Herpes B-positive status of one SHIV-infected animal Table 1 represents an overview of the Rhesus macaques used in this study. Table 2 shows the clinical results of the SHIV-positive rhesus macaques

### RNA isolation

Splenic tissue samples stored in RNALater^® ^(Ambion, TX) at -80C were thawed in 1.5 mL tubes on ice. Tissue was submerged in Trizol ™ (Invitrogen, CA) solution and RNA isolation was carried out paccording to the Trizol ™ Reagent manufacturer's recommended instructions. RNA was ethanol-precipitated, air-dried and re-suspended in 20 ul/sample of nuclease-free water. RNA quantity was measured via spectrophotometry followed by analysis with a Bioanalyzer 2100 (Agilent Technologies, CA)

### Custom made cDNA microarray SlidePreparation and hybridization

The gene library for the present project was commercially obtained from Research Genetics (Invitrogen, CA), containing 7489 genes, including 7019 known genes, 249 unknown genes and 110 expressed sequence tagged genes (ESTs). Superamine coated Telechem slides (Telechem Inc., OR) were used for printing the cDNA clones using 12 × 4 pin format, on a Virtek chip writer professional microarrayer in KemTek, Inc, MD. The printed slides underwent UV cross-linking, followed by post-processed by succinic anhydride treatment. The Micromax™ Tyramide Signal Amplification (TSA)™ Labeling and Detection Kit (PerkinElmer, Inc., MA) was used as directed by the manufacturer to determine relative gene expression of the collected samples. Custom-made reference RNA was prepared by combining aliquots of RNA from 33 normal Rhesus tissues and was used on every slide as the array controller, to check overall sensitivity of array printing, and to monitor reverse transcription, labeling and hybridization efficiency. Sample hybridization was carried out at 55°C for sixteen hours. A laser detection system was used (GenePix 4000b, Axon Instruments, CA) to scan the finished slides. Intensity of the scanned images was digitalized through Genepix 4.0 software (Axon Inc., CA).

### Microarray analysis

Data cleansing and statistical analysis was carried out using Genespring^® ^7.0 (Agilent Tech., CA). Local background was subtracted from individual spot intensity. Genes that failed this 'background check' in any of the eight given experiments were eliminated from further analysis. Each chip was next subjected to intra-chip normalization (LOWESS). The genes that varied most between control and treated sample sets were selected via *t*-test analysis. The *p*-value cutoff was set at 0.05. Four hundreds and thirty two genes were differentially expressed between SHIV -infected and control uninfected animals with p < 0.05.

The pattern of gene expression variability of the experimental set having reduced dimension was evaluated using principal component analysis (PCA) classifying SHIV positive and negative samples as the two variable classes [16].

### Real Time PCR

The t-test result was corroborated through real time polymerized chain reaction (Real-time PCR). A web-based primer designing tool was used to design the primers for the selected genes [17]. The specificity of each primer sequence was further confirmed by running a blast search. Reverse transcription and Real-time PCR reactions were carried out using reverse transcription kit (Invitrogen, CA) and Real-time PCR kit (Roche, IN), respectively. Each reaction with five technical duplicates was run in I-Cycler machine (Bio-Rad, CA). Each sample was also amplified against the house-keeping probe of the experiment: glyceraldehyde 3 phosphate dehydrogenase (GAPDH). The resultant cycle threshold data from each real-time-PCR 'run' was converted to fold-change using an established algorithm [5].

Quantitative and qualitative verification of the PCR product was accomplished by performing 1% agarose gel electrophoresis using SYBR Green I (Kemtek, Rockville, MD). Gel images were captured using PharosFX Molecular Imager system (Bio-Rad, CA) scanner and analyzed using Quantity One software (Bio-Rad, CA).

## Authors' contributions

EEC participated in the design of the study, carried out the microarray and real time PCR studies and participated in drafting the manuscript. RH participated in the design of the study, carried out the microarray data analysis, data mining and participated in drafting the manuscript. NC participated in the microarray data analysis and participated in drafting the manuscript. AP participated in the microarray and real time PCR studies.

SAM participated in the microarray and real time PCR studies. MJ conceived of the study, and participated in its design and coordination. All authors read and approved the final manuscript.
